# Supplementary material for: Single molecule array measures of LRRK2 kinase activity in serum link Parkinson’s disease severity to peripheral inflammation
Source: Mol Neurodegener. 2024 Jun 11;19:47. doi: 10.1186/s13024-024-00738-4 (PMC11167795; doi:10.1186/s13024-024-00738-4)
Supplement: Supplementary file 2 — Supplementary Material 2 [file 13024_2024_738_MOESM2_ESM.pdf]

Single molecule array measures of LRRK2 kinase activity in serum link Parkinson's disease severity to peripheral inflammation

Yuan Yuan[1,2#], Huizhong Li[1,2#], Kashyap Sreeram[1,2#], Tuyana Malankhanova[1,2], Ravindra Boddu[1,2], Samuel Strader[1,2], Allison Chang[1,2], Nicole Bryant[1,2], Talene A. Yacoubian[3], David G. Standaert[4], Madalynn Erb[4], Darren J. Moore[4], Laurie H. Sanders[1,5, 6], Michael W. Lutz[5, 6], Dmitry Velmeshev[7], Andrew B. West[1,2,3,5,7]**

[#]Denotes equal contributions

[1]Duke Center for Neurodegeneration and Neurotheraputics, Duke University, Durham, NC, USA

[2]Department of Pharmacology and Cancer Biology, Duke University, Durham, NC, USA

[3]Department of Neurology, University of Alabama at Birmingham, Birmingham, AL, USA

[4]Department of Neurodegenerative Science, Van Andel Institute, Grand Rapids, MI, USA

[5]Department of Neurology, Duke University, Durham, NC, USA

[6]Department of Pathology, Duke University, Durham, NC, USA

[7]Department of Neurobiology, Duke University, Durham, NC, USA

**Corresponding author. Tel: +1 919 684 1656; E-mail: Andrew.West@Duke.edu

Supplemental Figures 1-6

Supplemental Figure Legends 1-6

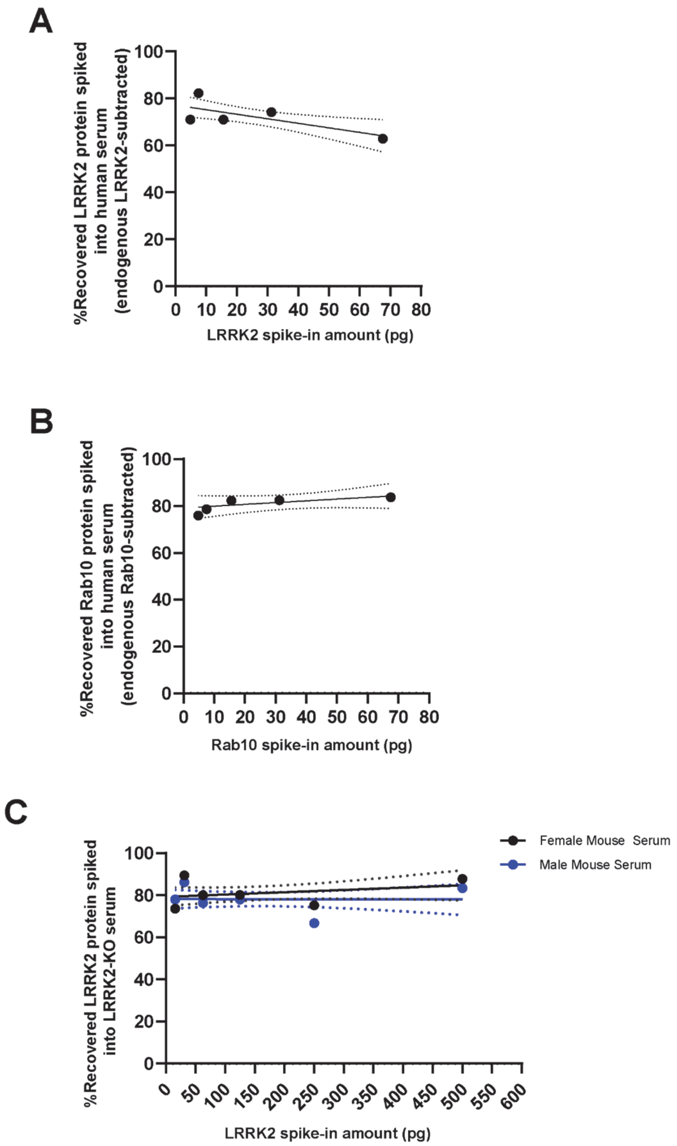

Supplemental Fig. 1. Spike and recovery results from recombinant proteins for LRRK2 and total Rab10 spikes into serum.

(A) Lineplots show the percent recovery of picogram quantities of recombinant LRRK2 protein or **(B)** Rab10 protein spiked into human serum samples, with the background levels of endogenous LRRK2 or Rab10 subtracted prior to the calculated recovery. Mean recovery across the indicated range was 72.2%±9% for LRRK2 and 81.8%±7% for Rab10. **(C)** Recovery of recombinant LRRK2 protein in male (blue) or female (red) sera from LRRK2 knockout (LRRK2$^{-/-}$) mice shows a similar recovery compared to human serum samples. Solid lines show mean linear regressions and dashed lines show 95% confidence intervals. Slopes in all plots are not significant according to correlation analysis.

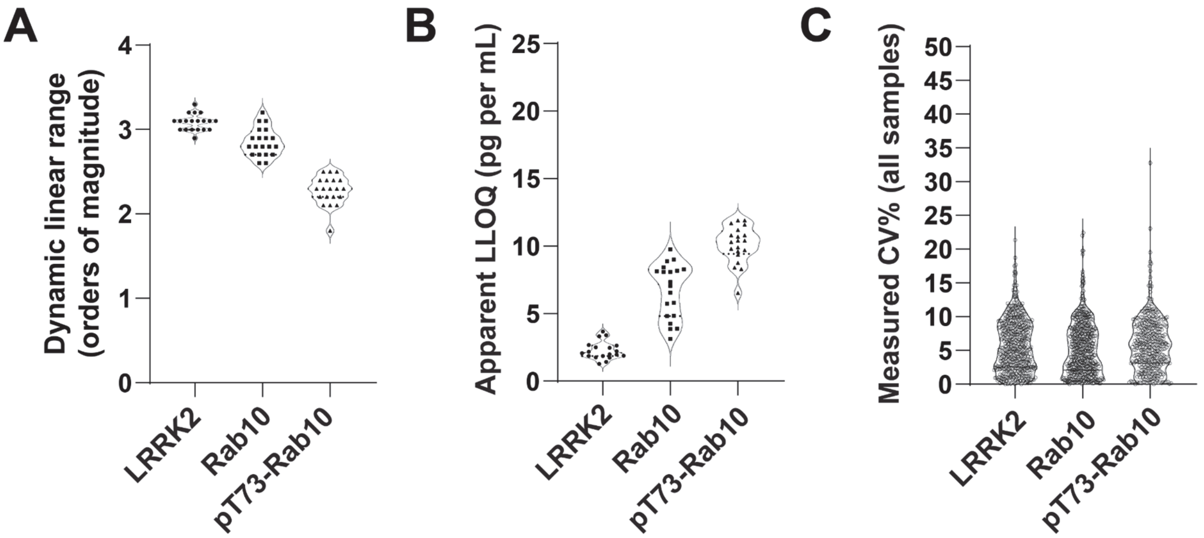

Supplemental Fig. 2. Good biomarker assay characteristics and reliability in biobanked human serum samples.

(A) Column graph shows measured assay dynamic range calculated from at least nineteen different runs per analyte (i.., LRRK2, Rab10, pT73-Rab10) on different plates. Mean observed dynamic range according to standard curves utilized in each run to resolve protein concentrations was consistent between runs and (in orders of magnitude) was calculated as 3.084 ± 0.095 S.D. for total LRRK2, 2.85 ± .16 S.D. for total Rab10, and 2.27 ± .17 S.D for pT73-Rab10.

(B) Column graph highlights good assay reproducibility with respect to lower limits of quantification (LLOQ) across different runs. Each dot represents the LLOQ on a different plate from a unique run. The empirically determined lower limit of quantification (in pg per mL) for each run was consistent between runs and calculated as 2.257 ± 0.65 pg/mL S.D. for total LRRK2, 6.709 ± 2.01 pg/mL S.D. for total Rab10, and 10.10 ± 1.37 pg/mL S.D for pT73-Rab10.

(C) Column graph provides evidence for excellent assay variance (CV, or coefficients of variability) across duplicated or triplicated human serum samples. Each dot shows the calculated CV calculated from one human serum sample. The average (mean) CVs are 5.816 ± 3.86 S.D. for measurements of total LRRK2, 5.361 ± 3.9 S.D. for measurements of total Rab10, and 5.906 ± 3.95 S.D for CVs related to the measures of pT73-Rab10.

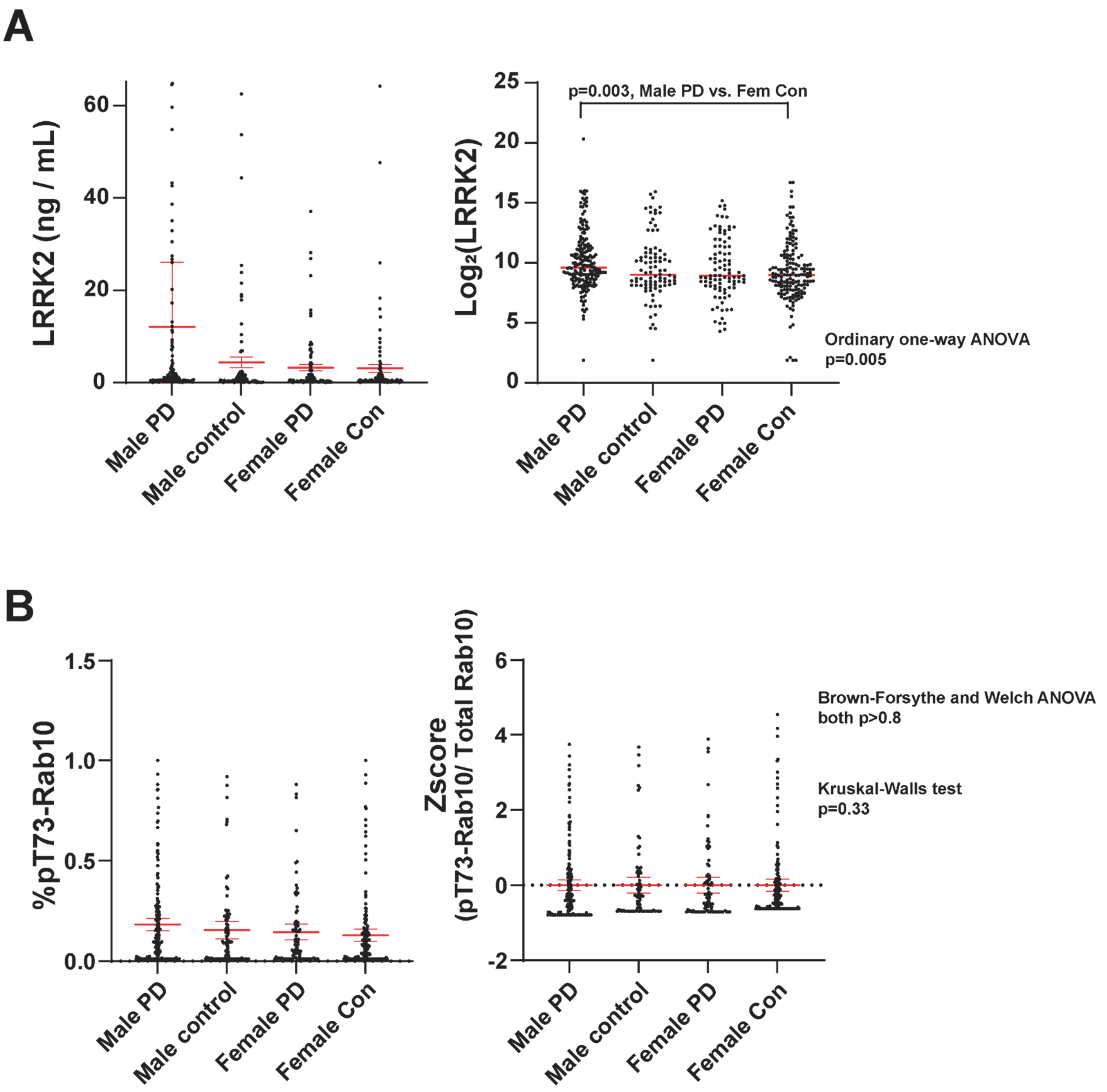

Supplemental Fig. 3. Serum LRRK2 and pT73-Rab10 to total Rab10 ratios are variable across subjects and similar in PD and control subjects.

(A) Scatter column plots show measured (unadjusted) LRRK2 levels in serum from male and female PD and control, and Log(2) transformation results that result in a normal distribution of LRRK2 expression in the groups. An ordinary one-way ANOVA is significant, with Tukey's post-hoc test demonstrating nominally increased levels of LRRK2 in male PD subjects versus control female subjects.

(B) Scatter column plots show measured (unadjusted) ratios of pT73-Rab10 to total Rab10 in serum from male and female PD and control. Attempts to transform the data into a normal distribution were not successful, and shown is a Z-score transformation that highlights the strata of subjects with very low (<0.09%) Rab10 phosphorylation (i.e., the ratio of pT73-Rab10 to total Rab10), in addition to some subjects with very high pT73-Rab10 levels. ANOVA test results do not support an overall difference in pT73-Rab10 ratios between groups. Together, these results suggest that LRRK2 and pT73-Rab10 levels in serum are unlikely to be useful for diagnostic purposes in the separation of PD cases from controls.

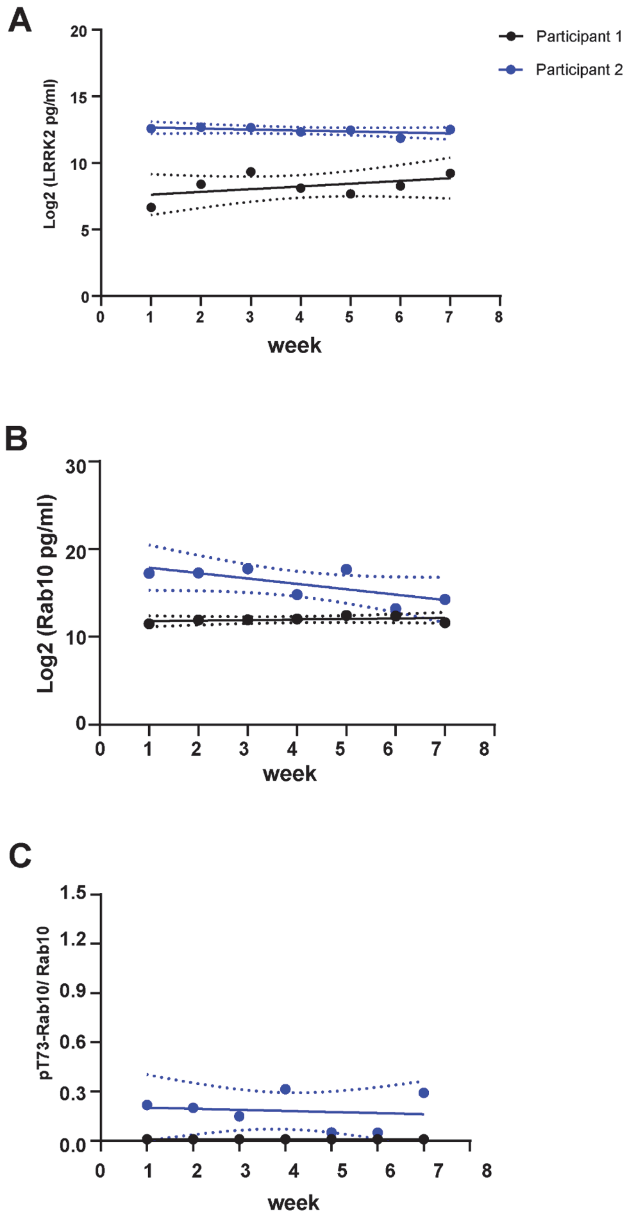

Supplemental Fig. 4. Longitudinal data (~2 months) for serum biomarkers from weekly serum depositions from 2 healthy control participants recruited in the PDBP cohort.

(A) Line graphs show LRRK2 expression sampling repeated with 1 week intervals for two control subjects recruited in the PDBP. Samples were run across different plates and randomized with final data curation prior to unblinding the samples (see Methods). **(B)** Total Rab10 levels over time, and **(C)** the ratio of pT73-Rab10 to total Rab10 over time. Solid lines show mean linear regressions and dashed lines show 95% confidence intervals. Slopes in all plots are not significant according to correlation analysis.

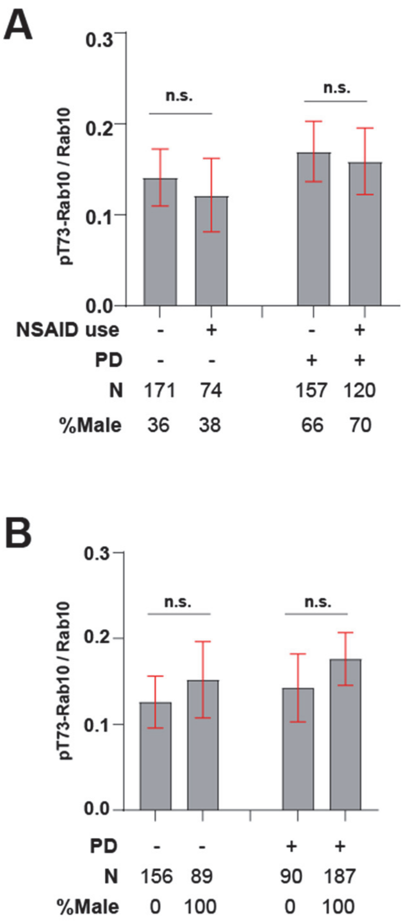

Supplemental Fig. 5. No interactions between the ratio of pT73-Rab10 to total Rab10 in NSAID use or sex as covariables.

Serum levels of total LRRK2 and pT73-Rab10/Rab10 ratios binned according to **(A)** usage of non-steroidal anti-inflammatory medication or **(B)** PD diagnosis and sex. The proportion of participants in each group diagnosed with PD is indicated. Column graphs show group means with 95% confidence intervals (C.I.) as red-colored error bars. p values are from Mann-Whitney tests for comparisons of the unequal group sizes, and n.s. is not significant ($p>0.05$).

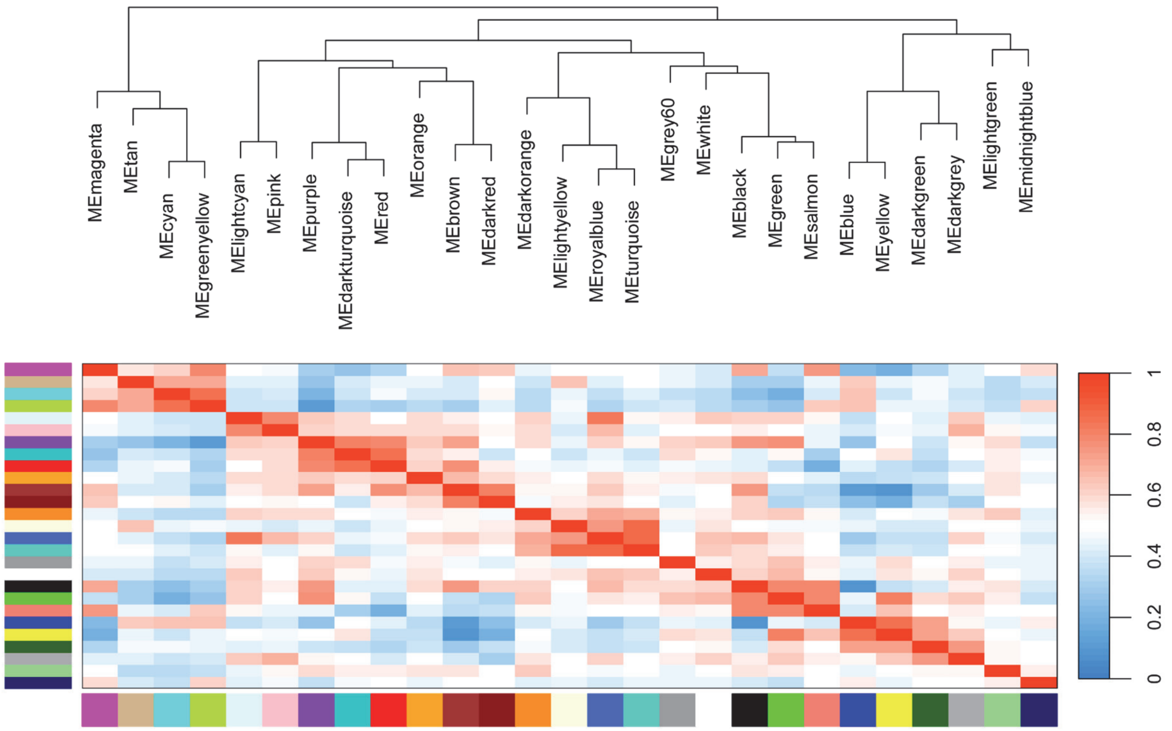

Supplemental Fig. 6. Hierarchical clustering and adjacency between modules identified in weighted correlation network analysis (WGCNA) of PD cases and controls (Table I).

The R library “WGCNA” was utilized to visualize modules of highly correlated genes from variance-stabilizing transformed transcriptomic data in whole blood samples. A total of 27 gene modules were produced. In order to study the relationships among the found modules, the eigengenes, or the first principal component of each module, were used to represent the gene expression profile and module similarity was quantified by correlating the eigengene values. The *plotEigengeneNetwork* function was used to generate a dendrogram and a corresponding heatmap. Hierarchical clustering of the eigengenes visualizes similarity. The modules of interest with respect to pT73-Rab10 to total Rab10 ratios, Purple and Light Cyan, are distantly related. The heatmap visualizes eigengene adjacency ($A_{IJ} = (1+cor(E_I, E_J))/2$), with higher values (red shades) indicating greater similarity. Purple and Light Cyan modules are similar to a moderate extent (adjacency = 0.63).
